# *Carthamus tinctorius* L. protects cerebral ischemia/reperfusion injury via arachidonic acid/p53-mediated apoptosis axis

**DOI:** 10.3389/fphar.2024.1504109

**Published:** 2024-12-24

**Authors:** Junren Chen, Liujun Wu, Xiaofang Xie, Cheng Peng

**Affiliations:** State Key Laboratory of Southwestern Chinese Medicine Resources, School of Pharmacy, Chengdu University of Traditional Chinese Medicine, Chengdu, China

**Keywords:** *Carthamus tinctorius* L., cerebral ischemia/reperfusion injury, metabolomics, arachidonic acid metabolism, P53 signaling pathway, apoptosis

## Abstract

**Introduction:**

Stroke is a debilitating disease and the second leading cause of death worldwide, of which ischemic stroke is the dominant type. *Carthamus tinctorius* L., also known as safflower, has been used to treat cerebrovascular diseases, especially ischemic stroke in many Asian countries. However, the underlying mechanisms of safflower in preventing ischemic stroke remains elusive. This study aims to elucidate the potential of safflower as a drug candidate for the prevention of ischemic stroke and to delineate its protective effects and potential mechanisms in a rat model of cerebral ischemia-reperfusion injury (CI/RI).

**Methods:**

The aqueous extract of safflower (AESF) was verified using HPLC-UV, HPLC-MS, and TLC. The inhibitory effect of AESF on platelet aggregation was detected *in vitro* and in zebrafish and mice. A CI/RI model in rats was established by middle cerebral artery occlusion and reperfusion to study the protective effect of AESF on ischemic stroke. 2,3,5-triphenyltetrazolium chloride, hematoxylin and eosin, and Nissl’s staining were employed to evaluate the pathological changes of brain tissue. In addition, metabolomics, ELISA, and Western blot were used to uncover the molecular alteration induced by AESF.

**Results:**

AESF significantly inhibited platelet aggregation *in vitro*, reduced the thrombogenesis in zebrafish, and prolonged clotting time in mice. In addition, AESF alleviated neurological dysfunction, cerebral oedema, cerebral infarct size, cerebral histopathological damage induced by ischemia-reperfusion, improved neuronal survival, increased serum levels of SOD and CAT, and decreased levels of iNOS and NO. Metabolomics revealed that AESF attenuated the metabolic disturbances in brain caused by I/R injury via regulating 38 metabolites particularly related to the arachidonic acid (AA) metabolism. Moreover, AESF elevated the serum levels of 6-keto-PGF_1α_, a pivotal metabolite of AA, downregulated the protein expression of p53, Bax, cleaved caspase-9, cleaved caspase-3, and cleaved caspase-8, and upregulated that of Bcl-2.

**Conclusion:**

AESF mitigated CI/RI through preventing platelet aggregation, alleviating oxidative stress, and suppressing apoptosis partially via modulating AA metabolism/p53-mediated apoptosis axis.

## Graphical Abstract

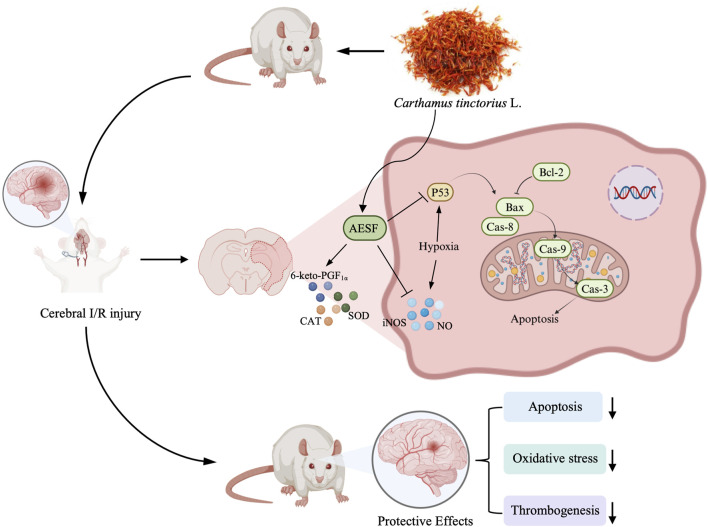

## 1 Introduction

Stroke is caused by a sudden rupture or blockage of a blood vessel in the brain that prevents blood from flowing to the brain, which mainly includes ischemic and hemorrhagic types (Saini et al., 2021). Stroke belongs to the category of neurological diseases, with extremely high mortality and disability rates, and is often accompanied by a variety of complications during its occurrence and development, which seriously endangers human health (Xu and Yao, 2021). Among the various subtypes of stroke, ischemic type accounts for about 87% (Ajoolabady et al., 2021); after ischemia, neuronal cells are unable to maintain normal transmembrane ionic gradients and homeostasis, which leads to oxidative stress injury or the release of large amounts of inflammatory factors, which exacerbates the damage of vascular endothelial and neuronal cells, leading to apoptosis and necrosis of brain tissue (Toman et al., 2019; Qin et al., 2019). For the treatment of stroke, the focus is on rapid reperfusion of the cerebral ischemia site by intravenous thrombolysis and endovascular thrombolysis within hours of onset, and interventional or thrombolytic therapy for recanalization of occluded cerebral vessels is the most effective way to treat ischemic stroke (Gomis and Davalos, 2014). Whereas, current thrombolytic therapies (e.g., alteplase) are associated with a range of risks, including hemorrhagic transformation, neurovascular injury and neuronal death (Durukan and Tatlisumak, 2007), and drugs targeting multiple pathophysiological events post cerebral ischemia have emerged as promising therapeutic strategies, but are still ineffective in reducing disability.

As a nuclear transcription factor, p53 modulates a series of physiological and pathological process including apoptosis, growth arrest, and senescence through regulating the expression of target genes in the context of genotoxicity or cellular stress (Hu et al., 2021). Remarkably, p53-mediated apoptotic signaling pathway was demonstrated to play a pivotal role in the development of stroke (Xia et al., 2021). During ischemic injury, rapid accumulation of p53 in brain tissue activates neuronal apoptosis through transcription-dependent and non-dependent procedures; p53 could also rapidly translocate to mitochondria and interact with Bcl-2 family proteins to activate the mitochondrial apoptotic program (Uzdensky, 2019). Both local and systemic metabolic changes can occur after cerebral ischemia, such as alterations in cellular energy metabolism pathways as well as the global stress response (Montaner et al., 2020). Metabolomics uncovered that disturbed metabolic pathways like arginine biosynthesis and alanine, aspartate and glutamate metabolism might be involved in the development of ischemic stroke in murine model, which mainly affect oxidative stress, energy failure, as well as apoptosis (Jia et al., 2021). Therefore, focusing on the alterations of metabolites during the pathogenesis of ischemic stroke and investigating the potential interactions between the metabolites and the p53-mediated apoptotic pathway could contribute to the exploration of new therapeutic strategies for ischemic stroke.

*Carthamus tinctorius* L. (safflower), one of the oldest crops, is widely used in the agricultural, industrial and medical fields (Zhang et al., 2016). In addition, safflower seeds and the pigment extracted from its flowers could be function as healthy vegetable oil and food additives, respectively, with great potential for development into functional foods (Tahir et al., 2020; Zhang et al., 2023). The critical hydrophilic metabolites of safflower include flavonoids, water-soluble heteropolysaccharides, alkaloids, saponins, and amino acids (Wu et al., 2021; Lu et al., 2021), among which flavonoids such as Hydroxysafflor yellow A (HSYA), Kaempferide, and Nicotiflorin might be the principal bioactive metabolites that have been reported to possess anti-inflammatory, antioxidant, anti-apoptotic, anti-cerebral ischemia-reperfusion injury (CI/RI) and cardiovascular protection effects (Xue et al., 2021). More importantly, safflower injectables display outstanding protective effects in cardiovascular and cerebrovascular diseases in clinic, especially stroke (Wang et al., 2018). Safflower extract alleviated cerebral infarction in rats by through suppressing inflammation and apoptosis via TNF-α/MAPK pathway and also ameliorated CI/RI by decreasing MMP-9 (Wang et al., 2020; Chang et al., 2020). However, there is a lack of research on safflower preventing ischemic stroke by attenuating p53-mediated apoptotic pathways, and fewer studies have focused on whether safflower prevents ischemic stroke by modulating key metabolites and pathways. Besides, the relationship between these key biomarkers and the pathways in which they are enriched and p53-mediated apoptosis has not been elucidated. Therefore, in the current study, we studied the effect of aqueous extract of safflower (AESF) in regulating the metabolite changes of CI/RI, and explored the regulation of AESF in mitigating apoptosis via p53 and arachidonic acid (AA) metabolism.

## 2 Materials and Methods

### 2.1 Identification and extraction of safflower

The dried flower of *Carthamus tinctorius* L. (safflower) from Sichuan Province, China was provided and identified by Prof. Jiang Chen of Chengdu University of Traditional Chinese Medicine. AESF was prepared as previously reported (Zhao et al., 2009). In brief, safflower was weighed and immersed with 15 times of double-distilled water for 30 min, then heated and extracted for 1 h. The filtrate was collected after filtration, and the residue was further extracted with 10 times of water and heated for 0.5 h. The filtrate was combined and concentrated for content detection and administration. 500 g of safflower was extracted and concentrated to yield 138.9 g of AESF.

### 2.2 HPLC-UV, HPLC-MS, and TLC analysis of AESF

#### 2.2.1 HPLC-UV analysis

**FIGURE 1:**
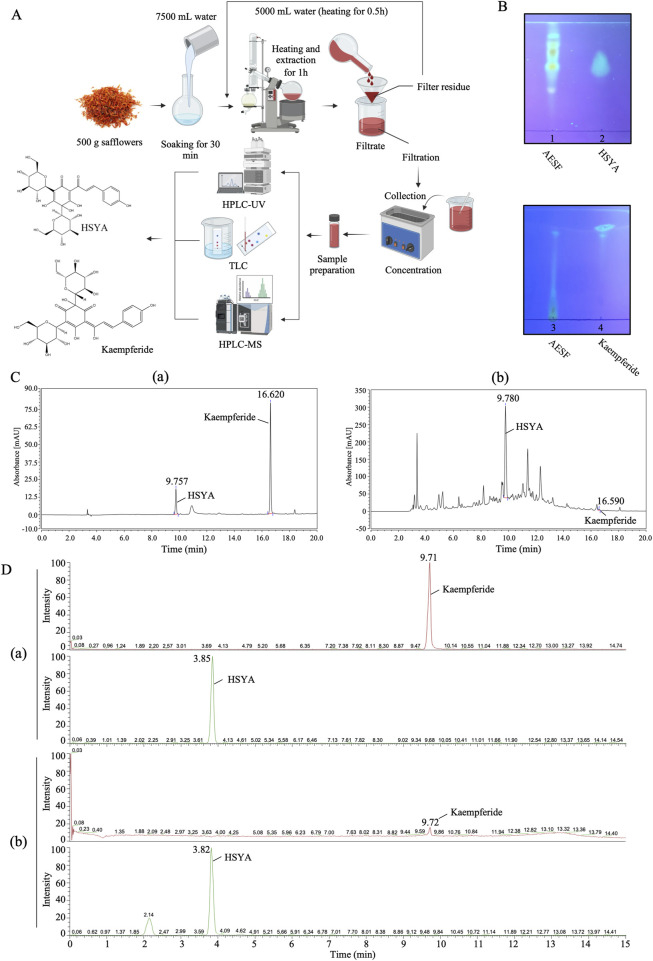
Qualitative and quantitative analysis of HSYA and Kaempferide in AESF. **(A)** The process of sample preparation for TLC, HPLC-UV, HPLC-MS analysis. **(B)** TLC analysis of HSYA and Kaempferide in AESF. **(C)** HPLC-UV analysis of standard HSYA and Kaempferide (a) as well as HSYA and Kaempferide in AESF (b). **(D)** HPLC-MS analysis of standard HSYA and Kaempferide (a) and quantitative analysis of HSYA and Kaempferide in AESF (b).

AESF was quantitatively analyzed by HPLC-UV (Figure 1A), and the test solution for the detection of HSYA and Kaempferide was prepared according to Chinese Pharmacopoeia (Chinese Pharmacopoeia Committee, 2020). 10 *μ*L test solution was injected into the chromatographic column (250 × 4.6 mm, 5 μm, Agilent) at 30°C, 254 nm and analyzed for 20 min. The mobile phase consisted of 0.4% phosphoric acid solution (eluent A) and acetonitrile (eluent B). The chromatographic separations were performed using gradient elution (0–15 min 5%–95% solvent B; 15–20 min 95% solvent B) with the mobile phase delivered at the flow rate of 1 mL/min. The content of the major metabolites HSYA and Kaempferide in AESF was quantitated by external reference method.

#### 2.2.2 HPLC-MS analysis

The preparation of test solution is the same as that mentioned in HPLC-UV analysis (100 × 2.1 mm, 3 *μ*m, Hypersil GOLD). The mobile phase consisted of 0.1% formic acid solution (eluent A) and acetonitrile (eluent B). The chromatographic separations were performed at 35°C using gradient elution (0–6 min 5%–35% solvent B; 6–12 min 35%–95% solvent B; 12–15 min 95% solvent B) with the mobile phase delivered at the flow rate of 0.3 mL/min. The electrospray ionization (ESI) of the mass spectrometer (MS) operated in negative-ion mode (ESI-). The parameters of ion source are as follows: negative ion: 3,500 V, sheath gas: 35 arb, aux gas: 15 arb, ion transfer tube temperature: 350°C, vaporizer temperature: 350°C. The masses of the investigated precursor, quantifier, and qualifier ions as well as the mass spectrometer parameter for HSYA and Kaempferide in AESF are provided in Table 1.

**TABLE 1:** The mass transitions and mass spectrometer parameters for HSYA and Kaempferide in AESF.

Analyte	Precursor ion (m/z)	Quantifier (m/z) Qualifier (m/z)	Collision energy (V)	Min Dwell time (ms)
Kaempferide	299.125	151.042 284	28.46 20.37	198.265
HSYA	611.3	325.155 491.238	29.77 24.08	198.265

#### 2.2.3 TLC analysis

0.5 g of AESF was added to 5 mL of 80% acetone solution, shaken for 15 min, and the supernatant was taken as the test solution. Appropriate amount of HSYA standard was weighed precisely, and 25% methanol was added to make a reference solution with a concentration of 0.6 mg/mL. Appropriate amount of Kaempferide was weighed precisely, and methanol was added to make a reference solution with a concentration of 1 mg/mL. For the detection of HSYA, 5 *μ*L of the test sample and the reference solution were spotted on the same silica gel H thin layer plate and the TLC plate was developed in the mobile phase: ethyl acetate-methanol-water-methanol (7:2:3:0.4, v/v). Then, the TLC plate was dried and viewed under UV light (365 nm). For the detection of Kaempferide, 5 *μ*L of the test sample and the reference solution were spotted on the same silica gel G thin layer plate and the TLC plate was developed in the mobile phase: chloroform-methanol-formic acid-water (12:3:1:0.3, v/v). Subsequently, the TLC plate was dried, sprayed with 1% aluminum trichloride in ethanol, incubated at 45°C for 5 min, and subjected to 365 nm UV light for detection.

### 2.3 Measurement of platelet aggregation rate

Rabbit heart blood was collected and added to a centrifuge tube containing 3.8% trisodium citrate solution (9:1 blood to sodium citrate ratio) and centrifuged at 800 r/min and 3,500 r/min to obtain platelet rich plasma (PRP) and platelet poor plasma (PPP), respectively. Adenosine-5 ′-diphosphate sodium salt (SIGMA, BCBP6676V) was employed as inducers of platelet aggregation. The rate of platelet aggregation was determined using a platelet aggregometer (Techlink Biomedical, AG800).

### 2.4 Measurement of thrombosis in zebrafish larvae

Zebrafish, obtained from China Zebrafish Resource Center (Wuhan, China), were maintained in a circulation system and were bred and propagated according to zebrafish book standards (Liao et al., 2021). 1-phenyl-2-thiourea (PTU) (SIGMA, BCBK8912V) was used to inhibit the formation of melanin in zebrafish larvae. AB line zebrafish larvae incubated to 3 days post fertilization (3 dpf) were randomly placed in 6-well plates and administered with 30 *μ*M aspirin or different concentrations (600, 800, and 1,000 *μ*g/mL) of AESF for 6 h, followed by 10 *μ*M arachidonic acid for 1 h. Subsequently, zebrafish were stained with o-anisidine staining solution, and the intensity of the heart erythrocyte staining and the caudal vein thrombus were observed using microscope (Leica Microsystems, Germany). Image-Pro Plus 6.0 software was used to analyze the heart erythrocyte staining intensity.

### 2.5 Measurement of clotting time

Clotting time assay was performed as previously reported (Bu et al., 2024). In brief, mice were orally administrated with 0.9 g/kg of AESF or 13.3 mg/kg of Aspirin for 5 consecutive days, and 1 h after the last administration, the tails of the mice were clipped for about 3 mm and allowed to naturally droop onto filter paper to record the bleeding time. In addition, blood was taken by inserting a glass capillary tube into the venous plexus of the mouse eye and placing a drop of blood on a clean slide. Thereafter, the clotting time was recorded by gently picking with a needle every 30 s until the blood could be picked up.

### 2.6 Establishment and treatment of CI/RI rat model

The male SD rats were purchased from SPF (Beijing) Biotechnology Co., Ltd. (Beijing, China), and were kept at 22°C ± 2°C with a 12 h light/dark cycle with *ad-libitum* food and water. SD rats were randomly divided into four groups: Sham-operated group (control group), middle cerebral artery occlusion (MCAO) group (model group), Aspirin group, and AESF group, with 25 rats in each group. Rats in Aspirin and AESF group were intragastrically administered with 89 mg/kg aspirin and 0.92 g/kg AESF once a day for consecutive 3 days before operation, while rats in the control group and model group were intragastrically administered with the same volume of saline solution. 1 h after the last administration, the CI/RI rat model induced by MCAO was replicated in each group (except for the control group) according to the modified Zea-Longa suture method (Ma et al., 2022).

Briefly, the rats were continuously anesthetized with isoflurane (21011401, RWD Life Science Co., Ltd.) and fixed supine on a homemade rat board. The neck was routinely prepared and disinfected with iodophor followed by deiodination with 75% alcohol. A longitudinal incision was made along the middle of the neck, and the tissue was bluntly separated with forceps to expose the right common carotid artery (CCA), internal carotid artery (ICA) and external carotid artery (ECA), and the vagus nerve was carefully stripped from the surface of the vessels. A slipknot was tied at the proximal end of CCA, and ICA was clipped with an artery clamp. Subsequently, ECA was double ligation and cut in the middle, and an arteriotomy hole was made at 3 mm from the vascular bifurcation of ECA. Then insert the silicone coated nylon thread (MSRC42B200PK50, RWD Life Science Co., Ltd.) from the opening to the ICA and open the arterial clamp, continue to insert the thread along the ICA into the middle cerebral artery (MCA), stop when there is resistance, secure the thread at the ECA with a suture, and loosen the slipknot in the CCA. The wound was applied with penicillin sodium powder, and the neck skin was sutured. After 1.5 h of ischemia, the thread was gently removed, and the ECA was ligated (Zhang S. et al., 2021). Animals in sham-operated group underwent the same operation without occlusion of middle cerebral artery.

### 2.7 Neurological deficit evaluation and cerebral blood perfusion detection

24 h after reperfusion, the neurological deficit of rats was evaluated as previously reported (Longa et al., 1989). Rats with scores of 0 and 4 were eliminated based on the Zea-Longa score, which represents no neurological damage and extremely severe neurological impairment, respectively. The rats with scores among 1 to 3 were selected for subsequent experiments. The cerebral blood flow in rats were detected with laser speckle imaging system (RWD Life Science Co., Ltd.) before surgery, 1.5 h after ischemia, and 24 h after reperfusion.

### 2.8 Sample collection and determination of brain edema

The brain tissue of rats was immediately removed and weighed, and then placed at 60°C for 24 h to determine the dry weight. Brain water content (%) = (wet weight - dry weight)/wet weight ×100%.

### 2.9 Measurement of cerebral infarct volume

Rats were sacrificed and their brains were carefully collected and immersed in a 2% TTC (BCCB241, Sigma) solution for 30 min at 37°C. The infarct areas were photographed and the infarcted area was analyzed by ImageJ software. Percentage of cerebral area = (infarct area/whole brain area) ×100%.

### 2.10 Serum Metabolomics analysis

The serum samples were collected. The methods of serum metabolomics analysis were as previously reported (Xing et al., 2024a). The exported mass spectrum data were visualized and analyzed by MassLynx V4.2 acquisition software.

### 2.11 Measurement of Serum biomarkers

The oxidative stress biomarkers were detected by Elisa kits (Elabscience Biotechnology Co. Ltd., China) according to the manufacturer’s protocols.

### 2.12 H&E Staining and nissl staining

The brain tissues were collected, and the 1 mm thick coronal sections near the ischemia-lateral optic chiasma were selected and fixed with 4% paraformaldehyde solution. The brain tissues were stained according to the conventional hematoxylin and eosin (H&E) and Nissl staining procedures. The pathological scores of H&E staining were assigned from 0 to 6 and the of Nissl bodies was analyzed using ImageJ (Xing et al., 2024b).

### 2.13 Western blot analysis

Western blot analysis was performed as described previously (Bu et al., 2024). ECL reagents (Thermo Fisher Scientific) and Imaging System (Tanon, China) were used to analyze the immunoblots. The antibodies used in this part were shown in Table 2.

**TABLE 2:** Antibody information used in the Western blot analysis.

Antibodies	Source	Production company	Catalog numbers
anti-Caspase-3	Rabbit	Cell Signaling Technology	#9662S
anti-Bcl-2	Rabbit	Cell Signaling Technology	#28150S
anti-p53	Mice	Cell Signaling Technology	#2524T
anti-Bax	Rabbit	Cell Signaling Technology	#2772S
anti-Caspase-8	Rabbit	Cell Signaling Technology	#4790T
anti-Caspase-9	Mouse	Cell Signaling Technology	#9508S
β-actin	Rabbit	Cell Signaling Technology	4970S
Anti-mice IgG, HPR-linked Antibody	Goat	Boster Biological Technology	BA1050
Anti-rabbit IgG, HPR-linked Antibody	Goat	Absin Biological Technology	Abs20040ss

### 2.14 Statistical analysis

The data were shown as mean ± SD and analyzed using SPSS 29.0. One-way ANOVA followed by the least significant difference (LSD) *post hoc* test was used to examine the multi-group comparisons. Student’s t-test was used for unpaired experimental data for the comparison between the two individual groups. A value of *p* < 0.05 was considered statistically significant.

## 3 Results

### 3.1 Quantification of flavonoids in AESF

The process of sample preparation for HPLC-UV, HPLC-MS, and TLC analysis, as well as the chemical structures of HSYA and Kaempferide were illustrated in Figure 1A. The results of TLC analysis found that the test substance chromatogram showed spots of the same color at the corresponding position of the chromatogram of the control substance, indicating that HSYA and Kaempferide are present in AESF (Figure 1B). The content of HSYA in AESF is 8.4 mg/g, while Kaempferide is barely detectable in AESF according to the results of HPLC-UV analysis (Figure 1C). In addition, the results of HPLC-MS analysis showed that the content of HSYA in AESF is 10.46 mg/g, and the content of Kaempferide is 9.6 μg/g (Figure 1D).

### 3.2 AESF inhibits platelet aggregation *in vitro* and *in vivo*

**FIGURE 2:**
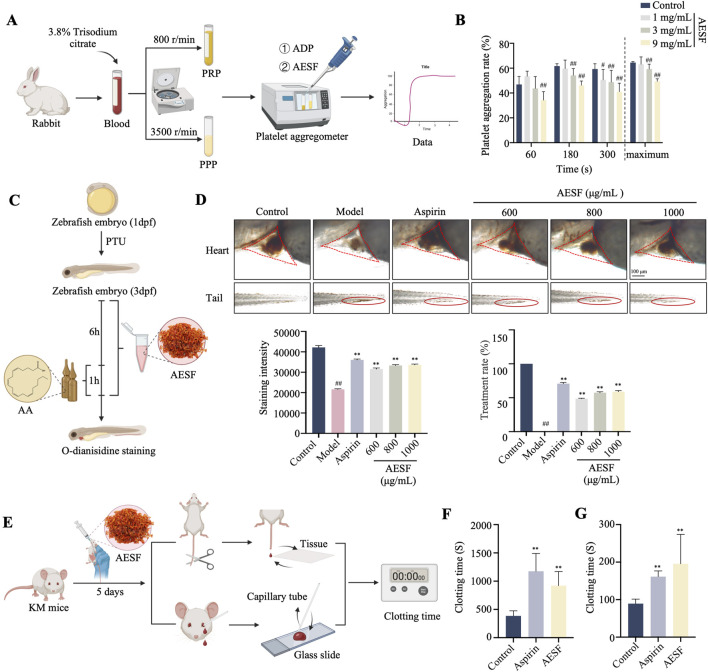
AESF inhibits platelet aggregation *in vitro* and *in vivo*. **(A)** Experimental procedure of the measurement of platelet aggregation rate. **(B)** The platelet aggregation rate in different measurement time points and the maximum platelet aggregation rate (n = 7). **(C)** Experimental procedure of the measurement of thrombosis in zebrafish larvae. **(D)** Representative images of the heart erythrocyte staining and the caudal vein thrombus of zebrafish (up), and the heart erythrocyte staining intensity and therapeutic rate of AESF on thrombosis (down) (n = 10). **(E)** Experimental procedure of the measurement of clotting time in mice. **(F, G)** The effect of AESF on clotting time under two different test methods (n = 6). ^#^ *p* < 0.05 and ^##^ *p* < 0.01 vs control group; ^*^ *p* < 0.05 and ^**^ *p* < 0.01 vs model group.

The experimental flowchart for *in vitro* anti-platelet aggregation of AESF is shown in Figure 2A. AESF at the concentration of 1, 3 and 9 mg/mL reduced the maximum aggregation rate induced by ADP *in vitro* compared with control group (Figure 2B). Subsequently, we measured the antithrombotic effect of AESF in zebrafish larvae and the experimental flowchart was portrayed in Figure 2C. As expected, AA intervention significantly decreased the intensity of the heart erythrocyte staining in zebrafish larvae and increased the caudal vein thrombus compared with control group, while AESF (at the concentrations of 600, 800, and 1,000 μg/mL) or Aspirin administration obviously reversed these changes induced by AA (Figure 2D). In addition, we detected the effect of AESF on clotting time in mice (Figure 2E). Compared with un-treated group, both AESF and Aspirin pre-treatment significantly prolonged the clotting time in mice under two different test methods (Figures 2F, G).

### 3.3 AESF mitigates nerve function deficit in I/R injury rats

**FIGURE 3:**
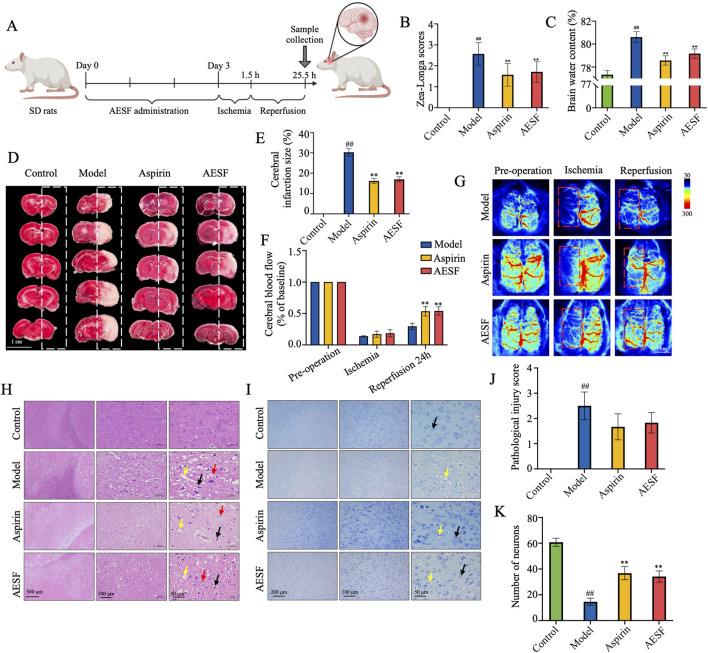
AESF alleviates CI/RI in rats. **(A)** Experimental procedure of the establishment of CI/RI rat model and the treatment scheme. **(B)** The change of Zea-Longa scores (n = 8). **(C)** The change of brain water content (n = 8). **(D, E)** TTC staining of the brain tissues of rats and the percentage of cerebral infarct volume (n = 8). **(F, G)** Representative images and the changes of cerebral blood flow in rats before surgery, 1.5 h after ischemia, and 24 h after reperfusion (n = 3). **(H–K)** H&E staining and Nissl’s Staining of brain tissues, pathological injury score, and number of neurons (n = 6). ^#^ *p* < 0.05 and ^##^ *p* < 0.01 vs control group; ^*^ *p* < 0.05 and ^**^ *p* < 0.01 vs model group.

After 3 days of AESF administration, the rats were subjected to 1.5 h of ischemia and 24 h of reperfusion (Figure 3A). Pre-intervention with AESF and Aspirin significantly reversed the increase of Zea-Longa scores (Figure 3B), decreased the brain water content (Figure 3C), and reduced cerebral infarct volume (Figures 3D, E) in rats induced by MCAO. In addition, the results of cerebral blood flow in rats showed that the CI/RI model in rat was successfully established and AESF treatment obviously increased the cerebral blood flow in rats 24 h after reperfusion (Figures 3F, G). Furthermore, the pathological results showed that AESF and Aspirin pre-treatment reversed the ischemic reperfusion injury in the brain tissue of the rats, as evidenced by a reduction in the number of crumpled neurons, a decrease in oedema and perineuronal cavities, a diminished necrosis of neurons, and a reduced neutrophil infiltration (Figures 3H, J). In addition, the results of Nissl’s staining showed that AESF and Aspirin reversed ischemia-reperfusion injury-induced laxity of neuronal cell arrangement, disappearance of nucleoli, and reduction of Nissl’s bodies in rat brain neurons (Figures 3I, K).

### 3.4 Effects of AESF on serum metabolites associated with CI/RI

**FIGURE 4:**
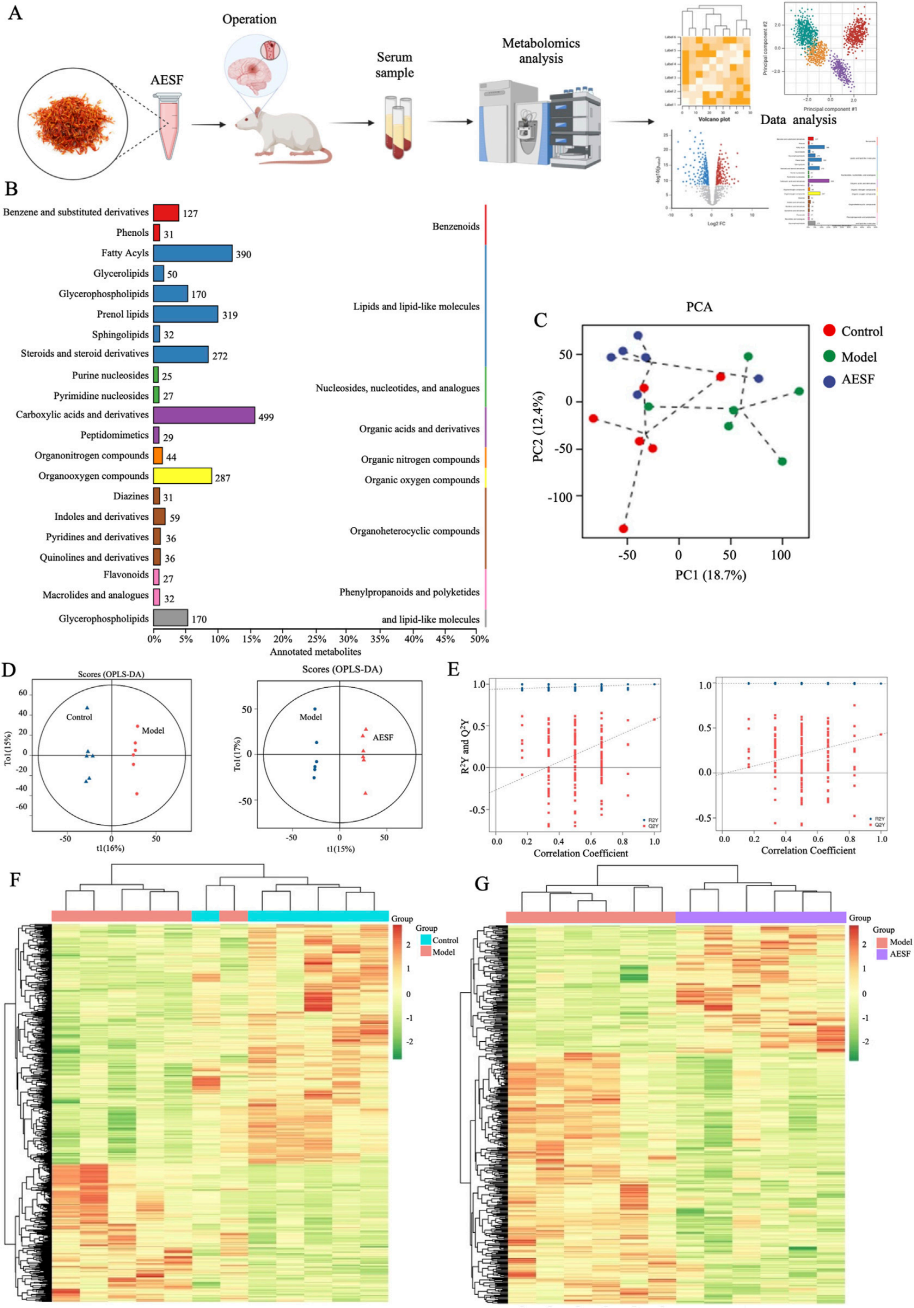
Measurement of metabolic profiles in serum of each group. **(A)** Experimental procedure. **(B)** The number and proportion of metabolites identified in each chemical classification. **(C)** PCA score plots of three groups. **(D)** Statistical validation of OPLS-DA model in positive (left) and negative (right) ion models. **(E)** Permutation test of the reliability of OPLS-DA model in positive (left) and negative (right) ion models. **(F, G)** Differential metabolite clustering heat map between control, model, and AESF groups, n = 6.

To further explore the mechanisms of AESF in protecting CI/RI, metabolomics analysis was used to detect the alteration of serum metabolites (Figure 4A). A total of 2,693 metabolites were identified, which were classified according to their chemical classification attribution information, including 499 carboxylic acids and their derivatives, 390 fatty acyls, 319 prenol lipids, 287 organooxygen compounds, 272 steroids and their derivatives, 170 glycerophospholipids, 127 benzene and substituted derivatives, and 59 indoles and their derivatives. In addition, some other metabolites with a relatively small percentage include organonitrogen compounds (1.30%), quinolines and their derivatives (1.07%), pyridines and their derivatives (1.07%), phenols (0.92%), and so on (Figure 4B). Principal component analysis (PCA) and orthogonal projections to latent structures-discriminant analysis (OPLS-DA), were used to map the serum metabolic profiles of rats and reflect the magnitude of variability between subgroups and between samples within groups. PCA analysis showed that the plasma samples of control rats were separated from those of model rats, and the serum samples of rats in AESF group were significantly separated from model rats, while demonstrated a trend towards the control group, indicating remarkable differences in serum metabolic profiles among control, model, and AESF groups (Figure 4C). Additionally, OPLS-DA analysis showed that Q^2^Y and R^2^Y values of model group and AESF group were 0.577 and 0.998, respectively (Figure 4D). Besides, the slope of the Q^2^Y fitted regression line was positive, and the R^2^Y of the model after replacement were higher than Q^2^Y (Figure 4E), which further proved significant differences in control vs model and model vs VESF group. Hierarchical clustering revealed that the expression of metabolites in model group was quite opposite to that in control and AESF group, that is, the metabolites with high expression in model group had low expression in control and AESF group (Figures 4F, G). These data implied that a model was established, and model group has obvious metabolic alterations compared with both control and AESF group.

**FIGURE 5:**
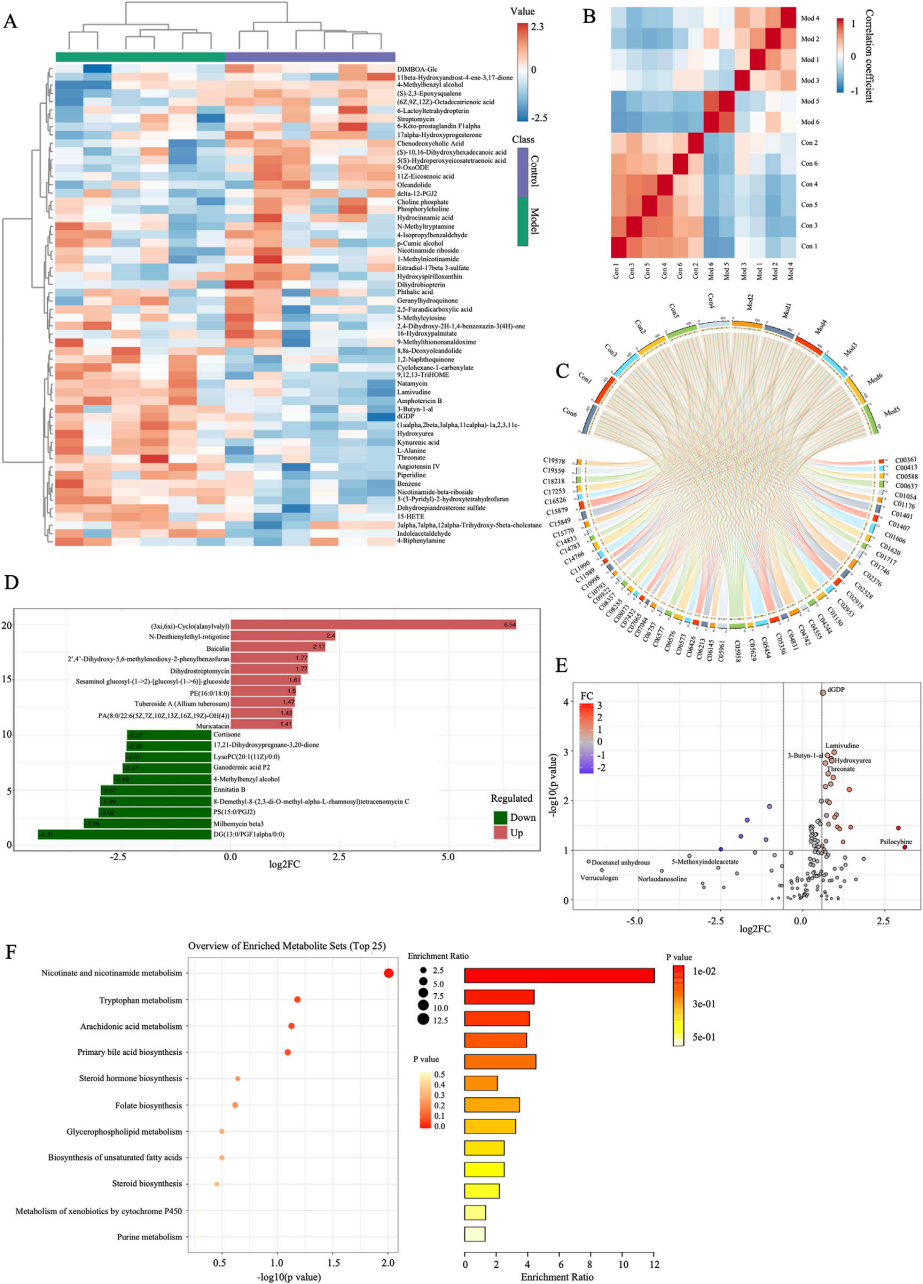
Screening of the potential markers in I/R. **(A)** Hierarchical cluster heatmap of serum metabolites between control and model groups. **(B)** Correlation Heatmap of metabolites in samples between model and AESF groups. **(C)** BMs and their contributions in control and model groups. **(D)** FC histogram of BMs in control and model group. **(E)** Volcano plot of the overall trend of BMs in metabolite content between model and normal groups. **(F)** Bubble diagram and histogram of the annotation results of BMs KEGG between model and normal groups, n = 6.

The variable importance in projection (VIP) values from the multivariate analysis of the OPLS-DA model allowed for an initial screening of metabolites that differed between groups. A total of 53 differential metabolites (DMs) were identified in the model group that were altered compared with control group, of which 21 were upregulated and 32 were downregulated, belonging to lipids and lipid-like molecules, organic oxygen compounds, organoheterocyclic compounds, benzenoids, organic acids and derivatives, organic nitrogen compounds, phenylpropanoids and polyketides, nucleosides, nucleotides, and analogues (Table 3). These DMs mainly belong to fatty acids, carboxylic acids and their derivatives, organic oxygen compounds, steroids and derivatives, etc., which might serve as potential biomarkers (BMs) for CI/RI. The results of hierarchical clustering heat map analysis illustrated that the metabolite tendency in model group was distant from control group (Figure 5A). In addition, the relationship between endogenous metabolites was identified by correlation analysis based on the pears-correlation coefficient, which verified that the correlation of the samples in model group was contrary to that of control group (Figures 5B, C). Among all the upregulated and downregulated metabolites, the top 20 BMs with the largest change in the differential multiple of quantitative information were shown by the histogram of the differential multiple. The most upregulated BM in the serum of rats in the model group is (3xi,6xi)-Cyclo (alanylvalyl), while the most downregulated DM is DG (13:0/PGF1alpha/0:0) (Figures 5D, E). The annotation results of KEGG for BMs were enriched and analyzed using ClusterProfiler, which indicated that CI/RI mainly affects nicotinate and nicotinamide metabolism (Figure 5F).

**TABLE 3:** BMs associated with cerebral I/R injury in rats.

Name	FC	P-value	VIP	Regulated	KEGG ID	Super class
Hydroxyurea	1.4926	0.0455	1.4519	up	C07044	Organic acids and derivatives
Lamivudine	1.5409	0.0105	1.7447	up	C07065	Nucleosides, nucleotides, and analogues
dGDP	1.2326	0.0375	1.4977	up	C00361	Phenylpropanoids and polyketides
L-Alanine	1.3406	0.0455	1.4258	up	C01401	Organic acids and derivatives
1,2-Naphthoquinone	1.4286	0.0428	1.5427	up	C14783	Benzenoids
3-Butyn-1-aL	1.3756	0.0500	1.4128	up	C06145	Organic oxygen compounds
Indoleacetaldehyde	1.6587	0.0426	1.6356	up	C00637	Organoheterocyclic compounds
9,12,13-TriHOME	2.1652	0.0274	1.6911	up	C14833	Lipids and lipid-like molecules
Threonate	1.5258	0.0443	1.4834	up	C01620	Organic oxygen compounds
Cyclohexane-1-carboxylate	1.1436	0.0490	1.4933	up	C09822	Organic acids and derivatives
15-HETE	1.3845	0.0312	1.5423	up	C04742	Lipids and lipid-like molecules
Nicotinamide-beta-riboside	2.2718	0.0060	1.9397	up	C03150	Organic oxygen compounds
Benzene	1.2296	0.0180	1.7260	up	C01407	Benzenoids
5-(3-Pyridyl)-2-hydroxytetrahydrofuran	1.5076	0.0211	1.7676	up	C19578	Organoheterocyclic compounds
Kynurenic acid	1.7454	0.0152	1.7139	up	C01717	Organoheterocyclic compounds
(1aalpha,2beta,3alpha,11calpha)-1a,2,3,11c-Tetrahydro-6,11-dimethylbenzo [6,7]phenanthro [3,4-b]oxirene-2,3-diol	1.8936	0.0165	1.7332	up	C19559	Benzenoids
Piperidine	1.1848	0.0393	1.5438	up	C01746	Organoheterocyclic compounds
Angiotensin IV	1.7794	0.0022	2.0946	up	C15849	Organic acids and derivatives
Natamycin	1.5535	0.0384	1.5416	up	C08073	Organic oxygen compounds
Dehydroepiandrosterone sulfate	1.5100	0.0304	1.5189	up	C04555	Lipids and lipid-like molecules
Amphotericin B	1.6896	0.0203	1.8160	up	C06573	Organic oxygen compounds
(S)-2,3-Epoxysqualene	0.4086	0.0019	2.0289	down	C01054	Lipids and lipid-like molecules
(6Z,9Z,12Z)-Octadecatrienoic acid	0.2021	0.0263	1.7842	down	C06426	Lipids and lipid-like molecules
4-Methylbenzyl alcohol	0.1558	0.0494	1.5478	down	C06757	Benzenoids
(S)-10,16-Dihydroxyhexadecanoic acid	0.6496	0.0282	1.6857	down	C08285	Lipids and lipid-like molecules
Streptomycin	0.7282	0.0346	1.6395	down	C00413	Benzenoids
11Z-Eicosenoic acid	0.3735	0.0319	1.7582	down	C16526	Lipids and lipid-like molecules
Dihydrobiopterin	0.8805	0.0003	2.2122	down	C02953	Organoheterocyclic compounds
p-Cumic alcohol	0.9307	0.0391	1.4465	down	C06576	Lipids and lipid-like molecules
Phosphorylcholine	0.7439	0.0294	1.7294	down	C00588	Organic nitrogen compounds
Chenodeoxycholic Acid	0.5597	0.0003	2.2740	down	C02528	Lipids and lipid-like molecules
6-Lactoyltetrahydropterin	0.8447	0.0442	1.5042	down	C04244	Organoheterocyclic compounds
9-OxoODE	0.4781	0.0074	2.0757	down	C14766	Lipids and lipid-like molecules
Choline phosphate	0.8618	0.0187	1.8353	down	C00588	Organic nitrogen compounds
5-Methylcytosine	0.9372	0.0127	1.7624	down	C02376	Organoheterocyclic compounds
16-Hydroxypalmitate	0.9103	0.0407	1.4847	down	C18218	Lipids and lipid-like molecules
Phthalic acid	0.9269	0.0351	1.5118	down	C01606	Benzenoids
2,4-Dihydroxy-2H-1,4-benzoxazin-3(4H)-one	0.9332	0.0173	1.6587	down	C15770	Organoheterocyclic compounds
11beta-Hydroxyandrost-4-ene-3,17-dione	0.9045	0.0333	1.5590	down	C07432	Organoheterocyclic compounds
17alpha-Hydroxyprogesterone	0.9010	0.0093	1.7675	down	C01176	Lipids and lipid-like molecules
2,5-Furandicarboxylic acid	0.9289	0.0341	1.5002	down	C20450	Organoheterocyclic compounds
6-Keto-prostaglandin F1alpha	0.9089	0.0055	1.8361	down	C05961	Lipids and lipid-like molecules
Hydrocinnamic acid	0.8760	0.0216	1.7079	down	C05629	Phenylpropanoids and polyketides
3alpha,7alpha,12alpha-Trihydroxy-5beta-cholestane	0.9287	0.0260	1.5502	down	C05454	Lipids and lipid-like molecules
4-Biphenylamine	0.9316	0.0426	1.4336	down	C10998	Benzenoids
DIMBOA-Glc	0.3625	0.0236	1.7882	down	C04831	Organic oxygen compounds
Estradiol-17beta 3-sulfate	0.6900	0.0462	1.6107	down	C08357	Lipids and lipid-like molecules
4-Isopropylbenzaldehyde	0.9239	0.0214	1.7755	down	C06577	Lipids and lipid-like molecules
delta-12-PGJ2	0.4368	0.0423	1.4700	down	C05958	Lipids and lipid-like molecules
N-Methyltryptamine	0.9292	0.0218	1.7498	down	C06213	Organoheterocyclic compounds
1-Methylnicotinamide	0.8894	0.0052	1.9144	down	C02918	Organoheterocyclic compounds
Nicotinamide riboside	0.8559	0.0089	1.9059	down	C03150	Organic oxygen compounds
5(S)-Hydroperoxyeicosatetraenoic acid	0.6147	0.0110	1.8993	down	C05356	Lipids and lipid-like molecules

**FIGURE 6:**
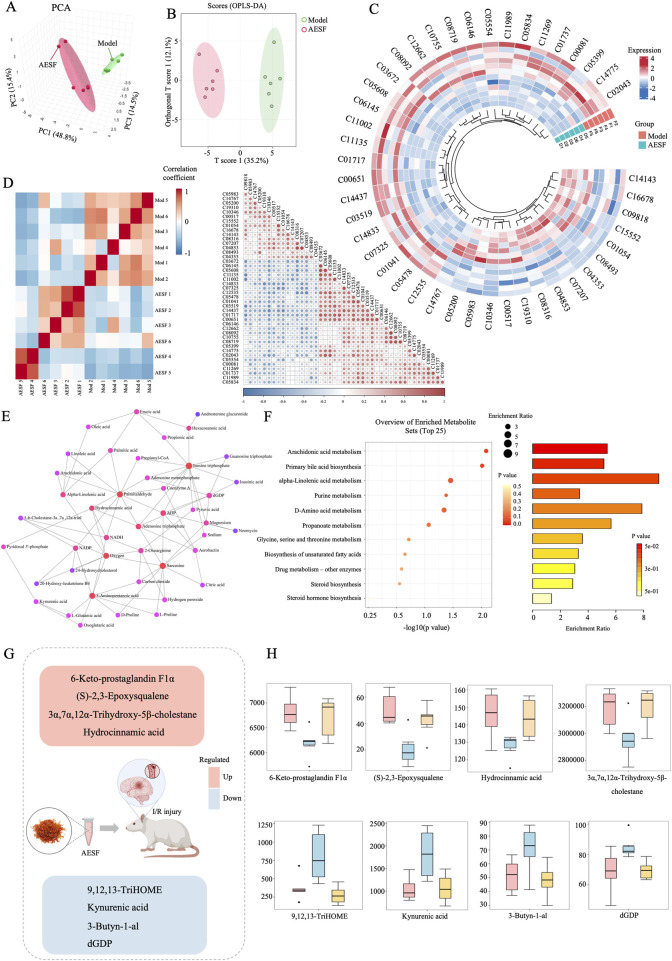
Differential metabolites involved in the regulation of AESF. **(A)** PCA score plots of DMs between model and AESF group. **(B)** Statistical validation of OPLS-DA model of DMs between model and AESF group. **(C)** Hierarchical cluster heatmap of serum metabolites between model and AESF groups. **(D)** Correlation Heatmap of metabolites in samples between model and AESF groups. **(E)** Metabolite-metabolite interaction network between model and AESF groups. **(F)** Bubble diagram and histogram of the annotation results of DMs KEGG between model and AESF groups. **(G, H)** The regulatory effect of AESF on important serum metabolites in CI/RI rats, n = 6.

A total of 38 DMs were identified in the AESF group that were altered compared with model group. Among them, 16 DMs were significantly downregulated, especially Androsterone glucuronide and Hydroxycinnamyl aldehyde, whereas 22 DMs were significantly upregulated especially (S)-2,3-Epoxysqualene and Propionyladenylate (Table 4). PCA analysis and OPLS-DA analysis showed that the DMs of AESF-treated rats were separated from those of model rats (Figures 6A, B), indicating remarkable differences in serum metabolic profiles between model and AESF groups. Moreover, the results from hierarchical clustering heat map analysis demonstrated that a significant distinction in the metabolite tendency could be observed between AESF group and model group (Figure 6C). Further correlation analysis also proved such findings (Figures 6D, E). Based on the above-mentioned potential DMs of model group, further screening of DMs between AESF group and model group was conducted. The annotation results of KEGG for DMs indicated that AESF treatment mainly affects AA metabolism (Figure 6F). A total of 8 DMs in serum of CI/RI rats were regulated after treatment with AESF, among which 4 DMs were obviously elevated and 4 DMs were remarkably decreased. These DMs belong to lipids and lipid-like molecules, phenylpropanoids and polyketides, organoheterocyclic compounds, and organic oxygen compounds (Table 5 and Figures 6G, H).

**TABLE 4:** DMs between AESF and model group in rats.

Name	FC	P-value	VIP	Regulated	KEGG ID	Super class
(S)-2,3-Epoxysqualene	2.0528	0.0121	1.8150	up	C01054	Lipids and lipid-like molecules
Zalcitabine	1.3784	0.0279	1.6862	up	C07207	Nucleosides, nucleotides, and analogues
all-trans-4-Oxoretinoic acid	1.4315	0.0280	1.6865	up	C16678	Lipids and lipid-like molecules
20-Hydroxy-leukotriene B4	1.2835	0.0241	1.7338	up	C04853	Lipids and lipid-like molecules
Erucic acid	1.2647	0.0277	1.6856	up	C08316	Lipids and lipid-like molecules
5-Aminopentanoate	1.1182	0.0379	1.6580	up	C00431	Organic acids and derivatives
Ectoine	1.0986	0.0471	1.5560	up	C06231	Organic acids and derivatives
9(S)-HODE	1.3700	0.0472	1.5894	up	C14767	Lipids and lipid-like molecules
5-Fluorouridine	1.2196	0.0240	1.7411	up	C16633	Nucleosides, nucleotides, and analogues
PE (18:0/0:0)	1.2370	0.0222	1.7501	up	C21484	Lipids and lipid-like molecules
6-Keto-prostaglandin F1alpha	1.0842	0.0332	1.6469	up	C05961	Lipids and lipid-like molecules
Hydrocinnamic acid	1.1224	0.0208	1.7595	up	C05629	Phenylpropanoids and polyketides
3alpha,7alpha,12alpha-Trihydroxy-5beta-cholestane	1.0791	0.0223	1.6980	up	C05454	Lipids and lipid-like molecules
Cortol	1.1259	0.0023	2.1697	up	C05482	Lipids and lipid-like molecules
alpha-Linolenic acid	1.0611	0.0100	1.8233	up	C06427	Lipids and lipid-like molecules
Cerebrosterol	1.0690	0.0295	1.6605	up	C13550	Lipids and lipid-like molecules
Propionyladenylate	1.6681	0.0448	1.5751	up	C05983	Nucleosides, nucleotides, and analogues
5-Amino-6-(5′-phosphoribitylamino)uracil	1.2028	0.0466	1.5203	up	C04454	Organic oxygen compounds
Adipyl-CoA	1.3932	0.0365	1.6305	up	C14143	Lipids and lipid-like molecules
5-Methylthiopentylglucosinolate	1.2431	0.0111	1.8911	up	C08401	Organic oxygen compounds
Palmitaldehyde	1.2844	0.0235	1.6779	up	C00517	Lipids and lipid-like molecules
1-[(4-Amino-3-methylphenyl)methyl]-5-(2,2-diphenylacetyl)-6,7-dihydro-4H-imidazo [4,5-c]pyridine-6-carboxylic acid	1.3920	0.0159	1.8515	up	C15552	Benzenoids
ITP	0.5675	0.0323	1.6784	down	C00081	Nucleosides, nucleotides, and analogues
L-Ascorbic acid	0.4637	0.0224	1.8417	down	C01041	Organoheterocyclic compounds
dGDP	0.8198	0.0032	2.0280	down	C00361	Phenylpropanoids and polyketides
3-Butyn-1-aL	0.6804	0.0262	1.7318	down	C06145	Organic oxygen compounds
Neomycin	0.3037	0.0217	1.7724	down	C01737	Organic oxygen compounds
4-Hydroxycinnamyl aldehyde	0.2959	0.0099	1.9757	down	C05608	Phenylpropanoids and polyketides
3-Methyldioxyindole	0.6322	0.0371	1.6992	down	C05834	Organoheterocyclic compounds
Androsterone glucuronide	0.0503	0.0493	1.6740	down	C11135	Lipids and lipid-like molecules
9,12,13-TriHOME	0.3420	0.0117	1.9568	down	C14833	Lipids and lipid-like molecules
Hexadecanal	0.3705	0.0393	1.6255	down	C00517	Lipids and lipid-like molecules
14,15-DHET	0.6316	0.0135	1.9415	down	C14775	Lipids and lipid-like molecules
Sarcosine	0.8207	0.0472	1.6225	down	C00213	Organic acids and derivatives
Kynurenic acid	0.5845	0.0183	1.8002	down	C01717	Organoheterocyclic compounds
Aerobactin	0.6457	0.0445	1.6074	down	C05554	Organic acids and derivatives
N-Acetyl-L-phenylalanine	0.5610	0.0075	1.9394	down	C03519	Organic acids and derivatives
2-Oxoarginine	0.8836	0.0401	1.6458	down	C03771	Organic acids and derivatives

**TABLE 5:** The effects of AESF on DMs in cerebral I/R injury rats.

Regulation (AESF vs. Model)	BM	Fold change	P-value	VIP	Super class	KEGG pathway
up	(S)-2,3-Epoxysqualene	2.053	0.012	1.815	Lipids and lipid-like molecules	Steroid biosynthesis; Sesquiterpenoid and triterpenoid biosynthesis; Biosynthesis of various alkaloid; Biosynthesis of plant secondary metabolites; Biosynthesis of terpenoids and steroids; Biosynthesis of plant hormone; Metabolic pathway; Biosynthesis of secondary metabolites
up	Hydrocinnamic acid	1.122	0.021	1.759	Phenylpropanoids and polyketides	Phenylalanine metabolism; Metabolic pathway; Microbial metabolism in diverse environments; Degradation of aromatic compounds
up	6-Keto-prostaglandin F1alpha	1.084	0.033	1.647	Lipids and lipid-like molecules	Arachidonic acid metabolism; Metabolic pathways
up	3alpha,7alpha,12alpha-Trihydroxy-5beta-cholestane	1.079	0.022	1.698	Lipids and lipid-like molecules	Primary bile acid biosynthesis; Metabolic pathways
down	9,12,13-TriHOME	0.342	0.012	1.957	Lipids and lipid-like molecules	Linoleic acid metabolism
down	Kynurenic acid	0.585	0.018	1.800	Organoheterocyclic compounds	Tryptophan metabolism; Metabolic pathways
down	3-Butyn-1-aL	0.680	0.026	1.732	Organic oxygen compounds	Butanoate metabolism
down	dGDP	0.820	0.003	2.028	Phenylpropanoids and polyketides	Purine metabolism; Metabolic pathway; Nucleotide metabolism

### 3.5 AESF suppresses apoptosis in CI/RI rats via regulating p53 signaling pathway

**FIGURE 7:**
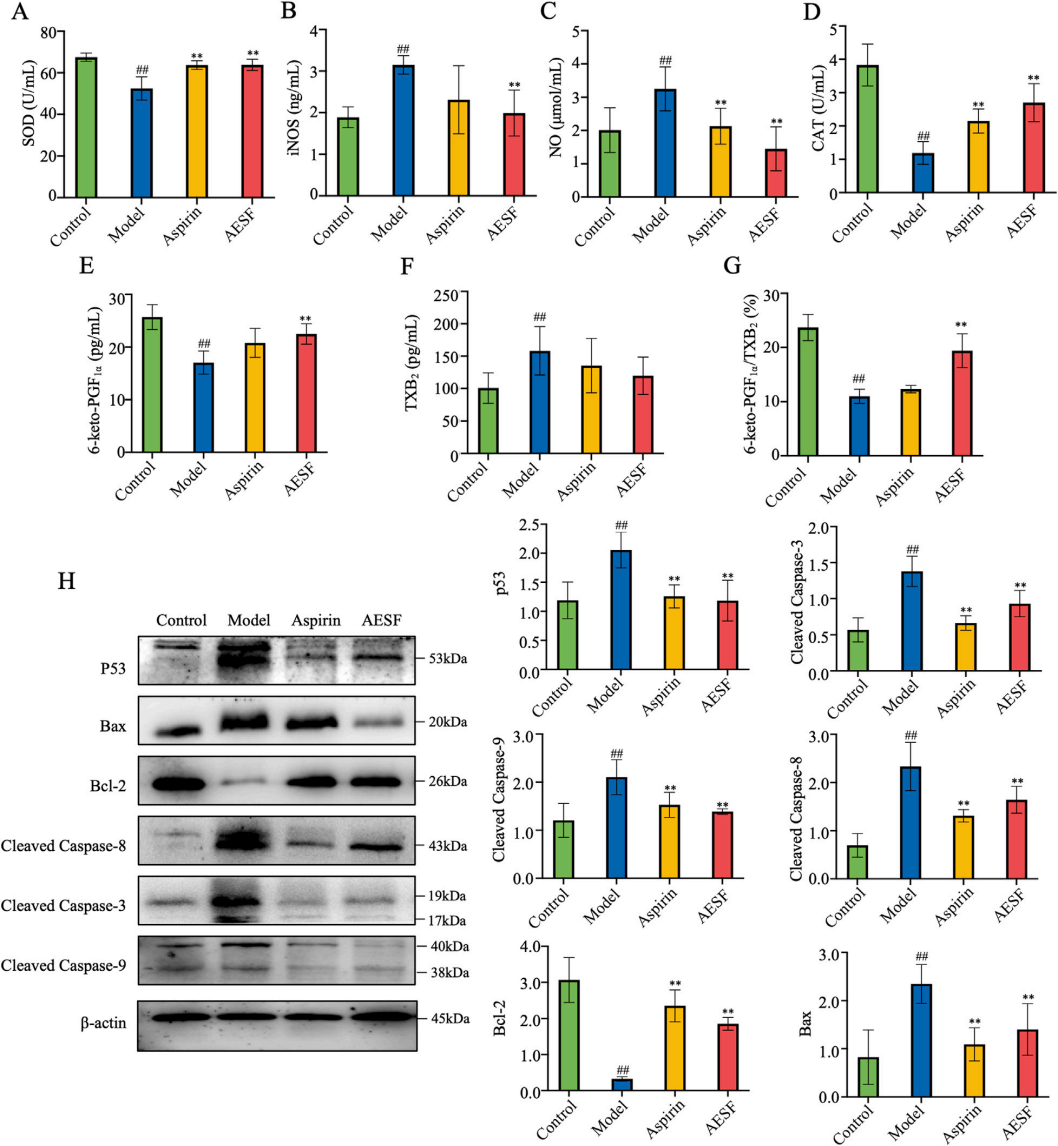
AESF alleviated CI/RI by inhibiting p53-mediated apoptotic signaling pathway. **(A–D)** The serum contents of SOD, iNOS, NO, and CAT (n = 8). **(E–G)** The serum contents of 6-keto-PGF_1α_ and TXB_2_ as well as the ratio of 6-keto-PGF_1α_/TXB_2_ in rats (n = 8). **(H)** The images of blot (left) and protein expression (right) of p53, Bax, cleaved caspase-8, cleaved caspase-9, cleaved caspase-3, and Bcl-2 in brain tissue of I/R rats (n = 3). ^##^ *p* < 0.01 vs control group; ^**^ *p* < 0.01 vs model group.

AESF and Aspirin could alleviate oxidative stress in CI/RI rats, which significantly enhanced the serum levels of SOD and CAT, while decreased that of iNOS and NO (Figures 7A–D). Moreover, metabolomics results showed that safflower treatment increased the levels of 6-keto-PGF_1α_, an important metabolite associated with AA. Therefore, we verified whether the serum levels of 6-keto-PGF_1α_ in rats were changed after AESF treatment. AESF administration increased the serum levels of 6-keto-PGF_1α_ compared with rats in model group and decreased that of TXB_2_, leading to elevated ratio of 6-keto-PGF_1α_/TXB_2_ (Figures 7E–G). AA metabolism has been reported to be associated with p53-mediated apoptotic signaling pathway (Hye Khan et al., 2016), we next investigated whether AESF attenuated CI/RI by modulating the p53 pathway. AESF treatment significantly reversed the upregulation of p53, Bax, cleaved-caspase-8, cleaved-caspase-9, and cleaved-caspase-3, as well as the downregulation of Bcl-2 in the brain tissue in rats induced by I/R injury (Figure 7H). Interestingly, Aspirin displayed similar effects to AESF on CI/RI rats.

## 4 Discussion

Safflower, belonging to the Compositae family, serves as a multifunctional cash crop in agriculture, industry and medicine (Zhang T. et al., 2021). Safflower has been reported to possess various bioactivities and therapeutic potential for kinds of cardiovascular and cerebrovascular diseases, such as cerebral ischemia and atherosclerosis (Li et al., 2022). Most current evidence indicates that flavonoids are the main material basis for the pharmacological activity of safflower (Xian et al., 2022). HSYA, as the most principal metabolite in AESF, was found to inhibit cell apoptosis and increase the Bcl-2/Bax ratio in ischemic penumbra of I/R-injured rats through activating PI3K/Akt/GSK-3β signaling pathway (Chen et al., 2013). In addition, HSYA also attenuated oxygen glucose deprivation-induced inflammatory responses in microglia by inhibiting the NF-κB signaling pathway and p38 phosphorylation (Li et al., 2013). Interestingly, several other bioactive flavonoids in safflower such as Nicotiflorin, kaempferol-3-O-rutinoside, and kaempferol-3-O-glucoside also exhibited promising therapeutic effect on ischemic stroke (Hu et al., 2017; Yu et al., 2013). Herein, we found that AESF significantly improved the CI/RI in rats through elevating the number of surviving neurons in ischemic penumbra and reducing the oxidative stress. However, considering the low dosage of AESF showed ineffectiveness against stroke and the rules of “3R” principles (replacement, reduction, refinement) in animal experiments, only 0.92 g/kg AESF was performed in the current study, and the dose-effect relationship of AESF in treating ischemic stroke will be discussed in the future research.

As a dynamic disease caused by multifactorial and multilayered injuries, the pathogenesis of ischemic stroke is complex and has not been fully elucidated. Increasing studies have confirmed that oxidative stress is the core pathological aspect of ischemia-reperfusion injury, and the overproduction of ROS and the over-depletion of antioxidant enzymes, such as SOD and glutathione peroxidase, are the important causes of oxidative stress injury (Zhang et al., 2022; Kelmanson et al., 2021). Therefore, mitigating oxidative stress injury plays a crucial role in the prevention and treatment of stroke. Safflower could improve CI/RI in rats through increasing SOD and reducing MDA (Zhang et al., 2020). CAT is the main enzyme system of endogenous resistance to peroxidation damage that can alleviate the damage of lipid peroxidation (Poprac et al., 2017). In the current study, AESF treatment upregulated the activity of CAT and the levels of SOD in CI/RI rats, which indicated that the protective effects of safflower on ischemic stroke might be conferred through alleviating oxidative stress. In addition, the concentration of NO increases explosively after CI/RI, which is involved in the process of delayed neuronal death and causes neurotoxicity (Zhang et al., 2024). iNOS is the main catalytic enzyme for the rapid increase of NO in the late stage of cerebral ischemic injury, and the aberrant expression of iNOS leads to the accumulation of NO, which interferes with the normal cellular metabolism and promotes the production of free radicals, thus aggravating CI/RI (Granger and Kvietys, 2015). Herein, we found that iNOS and NO were significantly increased in CI/RI rats, while AESF treatment reversed these changes.

In the present study, alterations of endogenous metabolites in serum of AESF-treated rats were identified by using metabolomic analysis. We found that AESF reversed I/R injury-triggered abnormalities in potential metabolite biomarkers, and these potential biomarkers are associated with biological pathways in ischemic stroke, such as AA metabolism, primary bile acid biosynthesis, alpha-linolenic acid metabolism, purine metabolism, D-amino acid metabolism, and propanoate metabolism. Importantly, AA metabolism was found to be the pathway with most significant differences. Various metabolic pathways of AA are closely related to the development of cerebrovascular diseases, and the role of its metabolites in I/R injury has been gradually confirmed (Kursun et al., 2022). Prostacyclin (PGI_2_) is a bioactive substance derived from AA catalyzed by COX-2, which is mainly synthesized and released from vascular endothelial cells, with the effects of antiplatelet aggregation, vasodilatation, and increased local blood flow (Blanco-Rivero et al., 2009). However, PGI_2_ has an extremely short half-life and is rapidly converted into the stable and biologically inactive 6-keto-PGF_1*α*_ *in vivo*. Thromboxane A_2_ (TXA_2_) is a bioactive factor that can promote vasoconstriction and platelet aggregation, which has a short half-life and is quickly converted to TXB_2_ (Colson et al., 2023). Under normal physiological conditions, PGI_2_ and TXA_2_ maintain a relatively balanced state to prevent thrombosis. However, ischemia and hypoxia disrupt the metabolism of AA that destroy the dynamic balance of PGI_2_ and TXA_2_, leading to intense vasoconstriction and occlusion, and exacerbating CI/RI (Yang et al., 2015). In this study, we found that AESF treatment obviously prevented the platelet aggregation *in vitro* and *in vivo*, and the ratio of 6-keto-PGF_1*α*_/TXB_2_ in serum of rats with CI/RI was significantly lower than that in the control group, while AESF could increase the level of 6-keto-PGF_1*α*_ and decrease the content of TXB_2_, suggesting that the protective effects of AESF on CI/RI might be associated with its ability to modulate AA metabolism.

Apoptosis is one of the main pathophysiological mechanisms of CI/RI that starts a few hours after the ischemic attack and mainly occurs in the ischemic penumbra (Tuo et al., 2022). The pathways of nerve cell apoptosis mainly include endoplasmic reticulum stress pathway, death receptor pathway and mitochondrial pathway (Tang et al., 2020). Ischemia induced apoptosis is mainly a mitochondria-centered process mediated by caspase family genes and Bcl-2 family genes. P53-mediated apoptotic pathway has been demonstrated to be implicated in the metabolism of AA (Hye Khan et al., 2016). The production of pro-apoptotic AA metabolites (e.g., Hydroxyeicosatetraenoic acids and leukotrienes) can activate mitochondrial pathways of apoptosis, promoting cytochrome c release and caspase activation (Ma et al., 2010; Li et al., 2024), while certain pro-survival AA metabolites (such as PGE_2_ from the COX pathway) are known to inhibit p53 activation and promote cell survival (Wang et al., 2015). This dual role allows AA metabolites to fine-tune the decision of cell between survival and apoptosis based on environmental cues and cellular stress levels. Herein, we found that AESF treatment downregulated the expression of p53 and elevated the AA metabolism in CI/RI rats. Additionally, p53 triggers apoptosis in ischemic stroke through interfering with the expression of pro- and anti-apoptotic proteins (Van Opdenbosch and Lamkanfi, 2019). Upon cerebral ischemic injury, Bcl-2 expression decreases and Bax expression increases, leading to activation of cysteine asparaginase, which triggers an apoptosis-dependent cascade of reactions (Creagh et al., 2003). Under normal physiological conditions, caspase-8, caspase-3, and caspase-9 are present as zymogens in the cell, whereas cerebral ischemic injury leads to an increase in oxygen free radicals, resulting in the disruption of the mitochondrial membrane structure and the activation of apoptotic signaling pathways, which activates these proteins and initiates an apoptotic response (Wang et al., 2016). Herein, we found that the expression of Bcl-2 in the brain tissue of rats in AESF groups was increased, and the expression of pro-apoptotic protein was significantly reduced, suggesting that AESF might alleviate CI/RI by modulating apoptosis pathway.

This study showed that safflower might attenuate ischemic stroke by modulating the AA metabolism/p53-mediated apoptosis signaling axis, which enriches the mechanism of action of safflower in the prevention and treatment of ischemic stroke. However, current evidence does not indicate a direct relationship between AA metabolism/p53-mediated apoptosis signaling axis and the protective effects of safflower against ischemic stroke, which suggests that further *in vitro* experiments like exploring the protective effects and mechanisms of the major metabolites in safflower against endothelial cell injury induced by oxygen-glucose deprivation and reperfusion are necessary. In addition, inhibiting the expression of p53 and crucial enzymes in AA metabolism *in vitro* and *in vivo* might contribute to validating the underlying mechanism of safflower in treating ischemic stroke.

## 5 Conclusion

In conclusion, AESF significantly relieves CI/RI in rats through preventing platelet aggregation, alleviating oxidative stress, and suppressing apoptosis via modulating AA metabolism and inactivating p53-mediated apoptosis signaling pathway. These findings reveal that targeting AA metabolism/p53-mediated apoptosis signal axis might be a possible strategy for the treatment of ischemic stroke, and safflower could be a candidate for ischemic stroke prevention.

## Data Availability

The original contributions presented in the study are included in the article/supplementary material, further inquiries can be directed to the corresponding authors.

## Abbreviations

AESF, Aqueous extract of safflower; AA, arachidonic acid; BMs, biomarkers; CCA, common carotid artery; DMs, differential metabolites; ECA, external carotid artery; H&E, hematoxylin and eosin; HSYA, Hydroxysafflor yellow A; I/R, ischemia-reperfusion; HPLC, high-performance liquid chromatography; ICA, internal carotid artery; MCAO, middle cerebral artery occlusion; OPLS-DA, orthogonal projections to latent structures-discriminant analysis; PCA, Principal component analysis; PGI_2_, prostacyclin; TXA_2_, thromboxane A_2_; UV-vis, ultraviolet and visible spectrophotometry; VIP, variable importance in projection.
